# Mismatch negativity reflects asymmetric pre-attentive harmonic interval discrimination

**DOI:** 10.1371/journal.pone.0196176

**Published:** 2018-04-25

**Authors:** Luise Wagner, Torsten Rahne, Stefan K. Plontke, Nico Heidekrüger

**Affiliations:** University Hospital Halle (Saale), Department of Otorhinolaryngology, Head and Neck Surgery, Martin Luther University Halle-Wittenberg, Halle (Saale), Germany; Universidad de Salamanca, SPAIN

## Abstract

**Objective:**

Western music is based on intervals; thus, interval discrimination is important for distinguishing the character of melodies or tracking melodies in polyphonic music. In this study the encoding of intervals in simultaneously presented sound is studied.

**Study design:**

In an electrophysiological experiment in 15 normal-hearing non-musicians, major thirds or fifths were presented in a controlled oddball paradigm. Harmonic intervals were created by simultaneously presented sinusoidals with randomized root frequency. Mismatch negativity (MMN) responses were measured with an EEG recording. The discrimination index was calculated in a psychoacoustic experiment.

**Results:**

A clear MMN response was found for the major third but not for the fifth. The neural generators were located within the auditory cortices. Psychoacoustically, no evidence was found that the subjects were able to detect the deviants.

**Conclusions:**

We conclude that pre-attentive discrimination of harmonic interval size is, in principle, possible in listeners without musical training although simultaneous presentation makes it harder to distinguish compared to non-overlapping intervals. Furthermore we see a difference in the response to infrequent dissonant stimuli in consonant standard stimuli compared to the opposite, rare consonant stimuli in dissonant standard stimuli.

## Introduction

Music perception in human beings is based on the detection of elements such as pitch, rhythm, dynamics, and timbre. In Western classical polyphonic music, synchronously sounding tones form so-called harmonic (or vertical) intervals. Physically, harmonic intervals can be described as ratios of the frequencies of two tones. The interval size between two notes of a diatonic scale can be considered as a multiple of semitones [1]. The interval is roughly the same at equal distances to the respective root note along the frequency axis [2,3]. The sound of a harmonic interval is rated as consonant if the frequency ratio is an integer value. Dissonance increases with decreasing frequency ratios; e.g., a minor second (16:15) is more dissonant than a major second (9:8), a major third (5:4), or a perfect fifth (3:2).

Cortical processing of musical sounds is influenced by musical training [4–8] and voluntary or involuntary attention [9]. Involuntary attention and pre-attentive cortical processing can be investigated with the mismatch negativity (MMN) event-related potential [10], which reflects the brain response to unexpected events and is usually studied using oddball paradigms. Therefore, a deviating stimulus is rarely presented in a series of repeated standard stimuli. Auditory MMN has successfully been recorded for deviances in pitch, duration, intensity, location, order, and other parameters of the stimuli [11, 12]. MMN is also elicited by abstract changes in auditory stimulation, such as language grammar and musical syntax violations (for a review, see [11]).

The so-called feature-detector model predicts asymmetric MMN amplitudes depending on the exclusion or inclusion of extra features in the deviant [13]. Larger amplitudes are observed if extra features are included in the deviant stimulus. If we would increase dissonance in the deviant interval, this model would predict larger MMN amplitudes than vice versa.

MMN studies have also focused on musical processing and perception [14]. Vuust et al. [15] elicited MMN using pitch mistuning, intensity, timbre, sound-source location, and rhythm cues. Harmonic intervals processing was assessed by Kölsch et al. [16] using major chords of three tones as standard stimuli. A marginally mistuned middle tone evoked an MMN as an indicator for pre-attentive processing of chords. Brattico et al. [17] examined the processing of minor, major chords and uncommon mistuned chords by musicians and non-musicians with MEG and found a more efficient discrimination for chords varying from conventional tonal music. In a controlled case study in a patient with acquired deafness to dissonance, Brattico et al. [18] found that there was a larger MMN amplitude to an infrequent dissonant interval (the major seventh) in a context of repeated consonant intervals (the major sixth) than there was to an infrequent consonant interval in a consonant context (e.g., the perfect octave replacing the major sixth). In the present study we investigated whether this asymmetry can also be found in normal hearing non-musicians.

Since these studies did not report a randomization of the absolute frequencies, no conclusions can be made about pre-attentive processing of interval size deviations independently of the absolute tone frequencies. Interval sequences based on root notes with constant frequency could also evoke an integration of those tones in auditory streams. The observed MMN would then have been evoked by a pitch deviation within a certain stream and not by the different interval size. Virtala et al. [19] avoided such a misleading observation by investigating major and minor dissonant and consonant chords and by varying the pitch of the standard major chords. Deviating minor, dissonant, or soft chords elicited a MMN. In the recent study the root notes are randomized.

The audiotory system seems to be sensitive to step direction as investigated by recording MMN [20]. Generally, it was later found that the auditory system has a preference for upward steps over downward steps, which can be seen in smaller psychoacoustic thresholds and larger MMN amplitudes for upward steps [21]. This asymmetry might also be expected regarding interval size processing, so that, e.g., infrequently presented fifths would evoke different MMN amplitudes than major thirds dependent on the frequently presented interval size.

The intent of this study was to objectively assess the pre-attentive processing of harmonic intervals in normal hearing subjects. We used harmonic intervals made of two sinusoidal tones with varying root frequencies and compared the elicited event-related potentials (ERPs) using an oddball paradigm. If deviations in interval size evoked a MMN, we hypothesized that harmonic interval discrimination is processed pre-attentively. To check if it is an MMN response dipoles are constructed and the location is checked according to [22].

## Materials and methods

### Subjects

Fifteen normal hearing (4PTA_0.5–4 kHz_ < 20 dB HL) listeners were recruited to participate in a prospective, controlled experimental study. All participants were between age 18 and 65 years and had no formal musical training. Written informed consent was obtained from all participants. The experimental procedures were in accordance with the guidelines in the Declaration of Helsinki and were approved by the ethics committee of the Martin Luther University Halle-Wittenberg. Every subject received monetary compensation for taking part in the study.

### Stimuli

Two simultaneously played sinusoidal tones were generated as wave files by MATLAB software (MathWorks Inc., Natick, MN, USA) applying a sample rate of 16 kHz. The frequency of the lower tone, i.e., the root note, was randomized and was set to either 300 Hz, 350 Hz, 400 Hz, 450 Hz, or 500 Hz. The frequency of the higher tone was adapted to the root note, forming either a major third with a frequency ratio of 5:4 or a perfect fifth with a frequency ratio of 3:2. The stimuli had a length of 100 ms including Hanning window-shaped ramps of 20 ms at the beginning and the end.

In an oddball paradigm, either the major third or the perfect fifth intervals were played infrequently as deviants, with a probability of 12.5%. If the fifths were used as deviants, then the major thirds were presented as standards, and vice versa. Each condition was presented in three consecutive blocks, with one block lasting about three minutes. In total, for each condition, 2000 standard and 250 deviant intervals were presented with a stimulus onset asynchrony of 400 ms in a passive listening task. Each deviant was followed by at least three standard intervals.

The stimuli were bilaterally presented through ER-3A insert earphones (Etymotic Research, Elk grove Village, Il, USA) with a sound pressure level of 70 dB. The stimulation process was implemented on a personal computer using STIM2 software (Compumedics, Singen, Germany).

### Data recording

#### Electrophysiological recordings

In the electrophysiological part of the study, the subjects were comfortably seated in a sound-attenuated room, where they watched a subtitled movie without sound and received instructions to disregard the presented auditory stimuli. They were not informed about the stimuli and the attendant features.

The subjects’ EEGs were continuously recorded with a Neuroscan SynAmps RT (Compumedics, Singen, Germany) AC coupled amplifier (sampling rate: 1000 Hz), using a 128-channel-electrode Standard BrainCap (EASYCAP GmbH, Herrsching, Germany) arranged on the scalp according to the extended International 10–20 system ("Report of the committee on methods of clinical examination in electroencephalography," 1958). A nose electrode was used as reference. The vertical electro-oculogram (EOG) was recorded with a bipolar electrode configuration on the left eye using additional electrodes. The EOG was later used for artifact reduction and to ensure that the participants were reading the subtitles of the movie. The electrode impedances were controlled before and after the measurement. Electrode impedances were kept below 20–30 kOhm.

#### Psychoacoustical measurements

Ten of the included fifteen subjects were available to participate in the psychoacoustic portion of the study. The same acoustical stimulation as in the electrophysiological portion was provided to the participants. The features of the interval stimuli were explained to them. After a training run, each subject’s task was to identify the major third or the fifth by pressing a button on a response key. The *fifth* and *major third* conditions were presented in an alternating order. The responses were recorded with Curry Neuroscan Software. The presentation of the respective blocks was made by an alternate sequence. A response was considered as “hit” if it occurred between 100 ms and 1000 ms after the target stimulus.

### Data analysis

The data analysis was conducted off-line with Curry Neuroscan Software. All data were baseline corrected before being off-line filtered using a 1 Hz to 20 Hz Butterworth bandpass filter (24 dB/octave) and a 50 Hz notch filter. Bad blocks due to muscular activity were removed manually. The EOG channels were inspected automatically for artifacts, and if the sample amplitudes were not between -150 μV and 150 μV, all channels were corrected using principal component analysis. For those participants with few blinking artifacts, the correction range was changed to between -100 μV and 100 μV. The first PCA component was always identified as blinking and then removed. Depending on the length of the blinking, the interval was 100 ms or 200 ms before and 200 ms or 300 ms after the detected artifact was corrected.

The preprocessed EEG data were segmented into epochs of 500 ms, with a 100 ms pre-stimulus beginning. Standard and deviant epochs were identified for both conditions and separately averaged. The two standard epochs recorded after a deviant and the last standard before a deviant were excluded. Epochs with a noise level larger than 1.6 times the average noise level were excluded.

The remaining epochs were further analyzed with MATLAB 2015 software, including the eeglab 13_6_5b toolbox. The nose electrode was used as reference. The base line correction was made with a 100 ms pre-stimulus interval. Bad channels with high impedances were excluded. The group mean waveforms were calculated for every electrode out of the averaged individual standard and deviant epochs for each subject. Difference waveforms for the *fifth* condition were calculated by subtracting the standard epochs of the *major third* condition (i.e., the responses to the fifths as standards) from the deviant epochs of the *fifth* condition; the difference waveforms for the *major third* condition were calculated by subtracting the standard epochs of the *fifth* condition (i.e., the responses to the major thirds as standards) from the deviant epochs of the *major third* condition. The latency of the MMN response was measured at the minimum of the group mean waveform at electrode Fz in a time window from 0 to 350 ms after stimulus onset. For each subject, the individual MMN amplitude was calculated as the mean voltage in a 40 ms time interval centered on the MMN peak latency of the group average waveform. The noise floor for statistical comparisons was calculated as mean amplitudes in a time interval with the same length as the interval around the MMN peak from 70 ms to 30 ms before stimulus onset.

The statistical analysis of the amplitudes was performed with SPSS20 software (IBM, Ehningen, Germany). The assumption of amplitude normality for both the MMN and the noise floor distributions was tested by the Kolmogorov-Smirnov test. The significance of the MMN amplitudes was tested with *t*-tests for paired samples, comparing the amplitude of the individual MMN in both conditions with the respective noise floor. The level of significance was reduced by the Bonferroni correction for multiple comparisons.

Source reconstruction was performed with BESA Research 6.1 software. One pair of symmetric dipoles and one dipole with free orientation were assumed and fitted on a 4-shell ellipsoidal with the LORETA algorithm. Sources were reconstructed for significant MMN amplitudes and also to compare the group average N1 response of the *major third* condition. The MMN source reconstruction used the interval from 100 to 200 ms after stimulus onset. Based on the zero crossings of the Cz potential of the major third condition, the interval for reconstructing the N1 sources was selected as 95 to 135 ms after stimulus onset (in a 40 ms time window around the minimum of the group average).

For the psychoacoustical part, the individual hit rates and false alarm rates were calculated for the two target intervals using Curry Software. The sensivity index d’ was calculated.

## Results

Fifteen normal hearing listeners (age range 20–65 years; mean age 31.2 years, 7 females and 8 males) participated in the experiment. All of them had no special musical training except for ordinary musical lessons in school. One EEG data set had to be excluded due to unexplainable technical artifacts.

**Fig 1:**
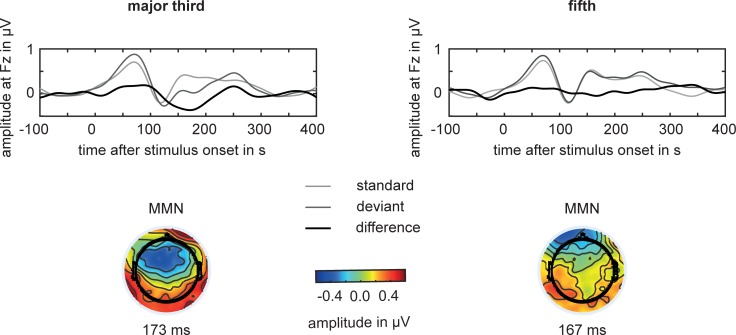
**Grand average ERP waveforms as elicited by ‘standard’ (light gray line) and ‘deviant’ (dark gray line) *major third* (left) or *fifth* (right) intervals at Fz.** The black lines show the difference waveforms between the ‘deviant’ and ‘standard’ waveforms. The contour maps show the distribution of response polarities on an average scalp at the latency of the MMN. Clear fronto-central negativity is only evoked by the major third deviants.

Fig 1 shows the group averages of the deviant and standard epochs as well as the difference curves. A clear MMN was observed only for the major third. The topoplots of the difference curves show a fronto-central localization of the MMN activity for the *major third* and no focused localization for the *fifth* condition.

The Kolmogorov-Smirnov test revealed a normal distribution of MMN amplitudes (p > 0.05) for all participants at the latency of maximal negativity in the group average. Also, the amplitudes of the noise floor were normally distributed. Thus, parametric tests were applied in further analyses.

For the *major third* condition, the paired *t*-test showed significant MMN amplitudes, -0.34 μV ± 0.32 μV at a mean latency of 173 ms, compared to the amplitudes 50 ms before stimulus onset (-0.01 μV ± 0.08 μV; *p* = 0.003). For the *fifth* condition, a paired *t*-test showed no significant MMN amplitudes at a latency of 167 ms (-0.02 μV ± 0.44 μV) compared to the potential 50 ms before stimulus onset (0.00 μV ± 0.09 μV; *p* = 0.83). A comparison between fifth and major third revealed no significant difference between the corresponding MMN amplitudes (*p* = 0.273).

**Fig 2:**
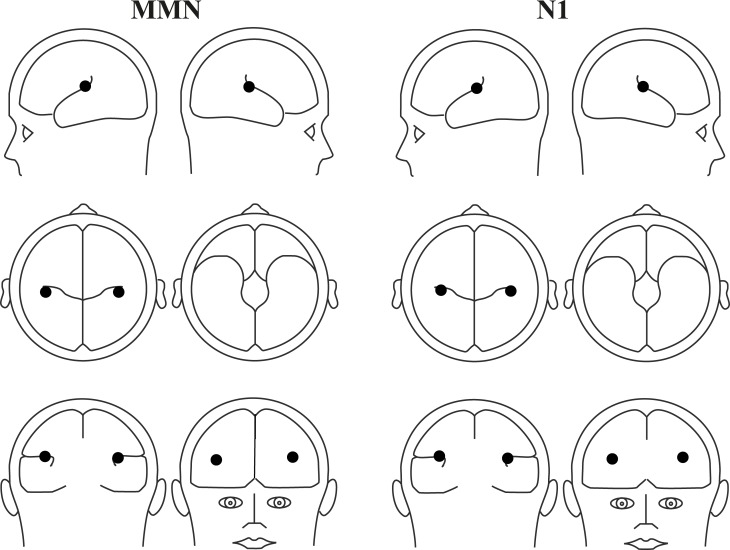
**Source reconstruction of the MMN (left) and N100 responses (right) for the *major third* condition using a standard head model.** Dipoles are located in the auditory cortices.

Fig 2 shows the source reconstruction results for the *major third* condition. The dipoles of the MMN were located within the auditory cortices, which have locations comparable to that of the N1 response.

**Fig 3:**
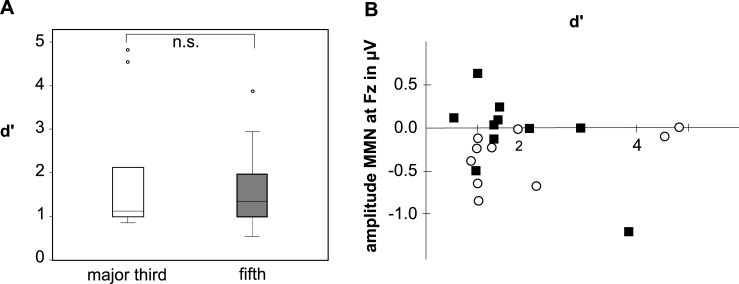
(A) Discrimination index as boxplots for the major third (white) and the fifth (gray). The difference is not significant (p > 0.05). (B) Individual MMN amplitude at Fz for the major third (white circle) and the fifth (black squares) as functions of the individual discrimination index d’.

Fig 3A shows the results of the psychoacoustic portion of the experiment. Three participants (30%) reached a discrimination score >1.5 for the fifth detection, and four participants (40%) reached that score for the major third detection. The mean discrimination indexes for the major third and fifth with its standard deviations were 1.66 ± 1.01 and 1.93 ± 1.51, respectively. This difference was not significant (p > 0.05). Fig 3B shows the MMN amplitudes at Fz as a function of the discrimination index. It is seen that the elicitation of a MMN response for the major third is independent of the individual psychoacoustic discrimination index and the amplitude does not increase with growing d’ (r = -0.48).

## Discussion

The results show that size differences in harmonic intervals evoke an MMN. If the interval size was infrequently reduced to a major third in a stream of fifths (*major third* condition), a clear response was evoked with a clear fronto-central distribution of negative response polarity and source dipoles within the auditory cortex. The response pattern and dipole sources are typical for a MMN response [22]. Thus, pre-attentive discrimination of harmonic interval differences is possible, in principle. Since an increase in interval size did not evoke significant MMN amplitudes (*fifth* condition) this automatic interval discrimination is asymmetrically.

This asymmetry is a little in contrast to the postulated preference of the auditory system towards upward steps as described in the introduction. Maybe the different root notes used in the present paradigm have masked the step directions. The asymmetry may also have been caused by the psychophysical feature of ‘disharmony’ since disharmony was increased by the major third more than it was by the fifth. As consonance is directly correlated to disharmony and is more important for the elicitation of the MMN than interval width [23], asymmetric MMN responses as observed in our study confirmed asymmetric pre-attentive discrimination of intervals. Another explanation for the asymmetry might be the different likelihoods of integrating intervals depending on their degree of consonance in one stream [24]. Also, different likelihoods of segregating low- and high-pitch streams would occur for fifths and major thirds [24]. A rare major third would evoke a gap in the high-frequency stream and, additionally, information in the root note stream. Thus, it is more likely to evoke an MMN with a major third than with a rare fifth causing reduced information within one single stream.

Psychoacoustically, the detection rate d’ of both interval size differences was with no significant difference between the conditions. However some participants surprisingly reported an easier detection of the fifths and some others for the thirds. The psychoacoustical results, however, do not reflect the individual interval discrimination skills, as this control experiment was not designed to measure those skills. For psychoacoustical measurements, longer stimuli and complex tones should be used rather than short sinusoidals [25].

Also, the root note frequency should be kept constant. Since a dissociation between MMN amplitudes and behavioral discrimination measures is prevalent in may paradigms (e.g. [26, 27]), our results emphasize the usefulness of objective measures to study cortical discrimination tasks.

None of the participants was musically trained. Although they could not detect the deviant intervals psychoacoustically in the presentation, pre-attentive detection was indicated by a significant MMN response.

We conclude that pre-attentive discrimination of harmonic interval size is, in principle, possible in listeners who lack musical training. Even if the target tones cannot be detected psychoacoustically, a MMN response is elicited.

## Supporting information

S1 Table The table shows the individual amplitudes of the subjects for the condition with major thirds or fifth as deviant at the latency of the MMN and the amplitudes of the noise floor before stimulus onset. (XLSX)

S2 Table The table shows the individual d‘ of each subjet for detecting the fifth or the major third. (XLSX)
